# Manipulating the Interferon Signaling Pathway: Implications for HIV Infection

**DOI:** 10.1007/s12250-019-00085-5

**Published:** 2019-02-14

**Authors:** Krystelle Nganou-Makamdop, Daniel C. Douek

**Affiliations:** 1 Institute of Clinical and Molecular Virology, University Hospital Erlangen, Erlangen, 91054 Germany; 2 Human Immunology Section, Vaccine Research Center, National Institute of Allergy and Infectious Disease, National Institutes of Health, Bethesda, MD 20892 USA

**Keywords:** Human immunodeficiency virus (HIV), Simian immunodeficiency virus (SIV), Type I interferon (IFN-I), Inflammation

## Abstract

During human immunodeficiency virus (HIV) infection, type I interferon (IFN-I) signaling induces an antiviral state that includes the production of restriction factors that inhibit virus replication, thereby limiting the infection. As seen in other viral infections, type I IFN can also increase systemic immune activation which, in HIV disease, is one of the strongest predictors of disease progression to acquired immune deficiency syndrome (AIDS) and non-AIDS morbidity and mortality. Moreover, IFN-I is associated with CD4 T cell depletion and attenuation of antigen-specific T cell responses. Therefore, therapeutic manipulation of IFN-I signaling to improve HIV disease outcome is a source of much interest and debate in the field. Recent studies have highlighted the importance of timing (acute vs. chronic infection) and have suggested that specific targeting of type I IFNs and their subtypes may help harness the beneficial roles of the IFN-I system while avoiding its deleterious activities.

## Introduction

Type I interferons (IFN-I) are cytokines abundantly produced during various immunological conditions and are known to play a pivotal role in the control of several viral infections. In humans, the IFN-I family is composed of 13 IFNα subtypes, IFNβ, IFNδ, IFNε, IFNζ, IFNτ, IFNκ, and IFNω (Pestka *et al.*^2004^), of which IFNα and IFNβ are the most predominant and best characterized. All type I IFNs bind with various affinities to their common receptor IFNAR, engagement of which triggers downstream transcription of a vast array of IFN-simulated genes (ISGs). Complete absence of IFN-I signaling by deletion of IFNAR in mice has been shown to enhance mortality from vesicular somatic virus, West Nile virus and lymphocytic choriomeningitis virus (LCMV) (Muller *et al.*^1994^). With repercussions for both innate and adaptive immune responses, the overall immunological consequence of IFN-I signaling is highly contextual and may vary across viral infections, bacterial infections, auto-immune disorders, and so on. Here, we focus on viral infections, specifically human immunodeficiency virus (HIV) infection during which IFN-I signaling via ISG expression in immune cells can lead to an antiviral state that interferes with several aspects of virus replication (Yan and Chen 2012).

## The Double Face of Type I IFNs

As early as a week after HIV infection, increased plasma levels of IFNα can be measured in infected individuals (Stacey *et al.*^2009^) and later on, elevated plasma levels of IFNα correlate with plasma HIV RNA and inversely with CD4 T cell count (Hardy *et al.*^2013^). Complementary data from animal studies have shown persistently higher ISG expression levels in simian immunodeficiency virus (SIV) infected rhesus macaques (Bosinger *et al.*^2009^; Jacquelin *et al.*^2009^; Sandler *et al.*^2014^). Importantly, IFN-I signaling resulting from SIV infection wanes during the transition from acute to chronic non-pathogenic infection in SIV natural hosts African green monkeys and sooty mangabeys, whereas a persistent response is associated with pathogenic infection and progression to acquired immune deficiency syndrome (AIDS) in experimental SIV infection of rhesus and pigtail macaques (Bosinger *et al.*^2009^; Jacquelin *et al.*^2009^; Harris *et al.*^2010^). Thus, these studies demonstrate a link between type I IFN signaling and pathogenic SIV infection.

## Beneficial Effects of IFN-I Signaling

The notion that IFN-I signaling can induce a protective antiviral state in the infected host yet at the same time be linked to pathogenic disease progression may be counterintuitive at first. Reported mechanisms by which IFN-I signaling interferes with virus replication in HIV infection include, among others, expression of a specific set of ISGs referred to as restriction factors, that inhibit the virus at various stages of replication such as cell entry (TRIM5α), reverse transcription (SAMHD1 and APOBEC3), nuclear entry and integration (MX2), translation and assembly (SLFN 11 and GBP5) and budding (Tetherin) (Soper *et al.*^2017^). With over 300 known ISGs (Schoggins *et al.*^2011^; Liu *et al.*^2012^), the increasing number of reported restriction factors with functional effects observed *in vitro* and *in vivo* (Soper *et al.*^2017^; El-Diwany *et al.*^2018^) altogether reinforce the knowledge that IFN-I signaling is critical to the suppression of HIV during early stages of infection. Sandler demonstrated that protection from a systemic SIV infection upon intrarectal challenge was achieved in rhesus macaques by intramuscular administration of IFNα and was associated with increased upregulation of ISG including restriction factors (Sandler *et al.*^2014^). Likewise, upregulation of ISGs in vaginal cell suspensions was shown to associate with protection from simian/human immunodeficiency virus (SHIV) infection in rhesus macaques that had received vaginal IFNβ administration (Veazey *et al.*^2016^).

## Detrimental Effects of IFN-I Signaling

Aside from restriction factors, other ISGs enhance pathogen associated molecular pattern (PAMP) detection and IFN signaling. For example, in response to viral PAMPs, IFN-I produced by plasmacytoid dendritic cells (pDCs) induces expression of the transcription factor IRF7 in neighboring cells which results in systemic expansion of IFN-I signaling. Similarly, other ISGs such as cGAMP and IFI16 all contribute to a persistent production of IFN-I (Bosinger and Utay 2015). The constant engagement of IFN-I signaling is thought to be at least in part responsible for the detrimental effects of IFN-I. Several studies have reported an association between IFN-I signaling and enhanced CD4 T cell depletion, potentially mediated through increased apoptosis, increase of CCR5-expressing HIV target cells or suppression of thymic output (Utay and Douek 2016). Moreover, studies on LCMV have shown that chronic IFN-I signaling is associated with immune suppression characterized by reduced antigen-specific T cell responses (Teijaro *et al.*^2013^; Wilson *et al.*^2013^), reduced T cell proliferation (Marshall *et al.*^2011^) and increased CD8 T cell exhaustion (Teijaro *et al.*^2013^; Wilson *et al.*^2013^). In HIV disease, type I interferon signaling is a major driver of immune activation, one of the strongest predictors of HIV disease progression to AIDS and non-AIDS morbidity and mortality (Hunt 2012). Collectively, these findings raised considerable interest in the potential therapeutic benefits of blocking IFN-I during HIV infection.

## Lessons Learned from Manipulating IFN-I Signaling *In Vivo*

### Enhancing IFN-I Signaling

Increased understanding of the opposing effects of type I IFN justifies the rationale for HIV/SIV studies aiming to enhance or block IFN-I signaling. In a considerable number of clinical studies (reviewed in Bosinger and Utay 2015), IFNα was administered subcutaneously, intramuscularly or orally to HIV infected persons not on antiretroviral therapy (ART). While some studies reported attenuation of CD4 T cell decline, lower HIV burden and opportunistic infections, others reported no significant improvements. These conflicting reports in conjunction with the efficacy of combinational ART underlined the impracticability of IFNα monotherapy for HIV treatment. A second wave of clinical studies investigated IFNα as supplement to ART. Data from over a dozen of studies showed no significant improvement of CD4 T cell recovery or clinical outcome, refuting a beneficial effect of IFNα as supplement to ART (Bosinger and Utay 2015). In a study by Massanella, pegylated interferon-α2a treatment of HIV infected persons on ART decreased absolute number of CD4 and CD8 T cells (Massanella *et al.*^2010^). More recently, studies largely conducted in animal models highlighted the importance of discriminating between three major HIV disease states: acute infection (where antiviral factors are upregulated), chronic ART-untreated infection (where both permanent low-level ISG expression and CD4 T cell depletion are maintained), and chronic ART-treated infection (where CD4 T cell recovery occurs and inflammation is at its lowest—albeit not normalized). Systemic administration of IFNα during rectal challenge transiently increased ISGs and prevented SIV acquisition in rhesus macaques (Sandler *et al.*^2014^) whereas administration of pegylated IFN-α2a to chronically SIV-infected ART-treated macaques upregulated ISGs with no effect on CD4 T cell depletion, immune activation or virus reservoir (Palesch *et al.*^2018^). Thus, in the acute stage of HIV infection, enhancing IFN-I signaling may be clinically beneficial but in the chronic stage there is to date no tangible evidence supporting such approach. In fact, the immunological consequences of a persisting immune activation would advocate for a suppression rather than enhancement of IFN-I signaling during chronic HIV infection.

### Blocking IFN-I Signaling

Blocking IFNAR during acute SIV infection *in vivo* increased SIV plasma viremia (Sandler *et al.*^2014^; Carnathan *et al.*^2018^), supporting the importance of IFN-I signaling for control of the infection during acute stage. In contrast, reduced inflammation and improved viral clearance was observed after blockade of IFN-I signaling with an anti-IFNAR antibody in murine LCMV infection (Teijaro *et al.*^2013^; Wilson *et al.*^2013^). Recently, two independent studies showed that administration of anti-IFNAR antibodies to ART-suppressed, HIV-infected humanized mice resulted in reduced immune activation and lowered reservoir size (Cheng *et al.*^2017^; Zhen *et al.*^2017^). The results of these mouse studies encouraged the idea that blocking IFN-I signaling during chronic HIV infection may help improve clinical outcome. In chronically SIV-infected rhesus macaques, systemic administration of a long half-life IFN-I antagonist significantly decreased ISGs with no impact on plasma virus load, immune activation or virus reservoir, irrespective of ART (Nganou-Makamdop *et al.*^2018^). With no obvious adverse effects, it remains to be determined what benefits will be gained from blocking IFN-I signaling during chronic HIV infection. Reduced T cell activation and virus reservoir achieved in humanized mice treated with anti-IFNAR blocking antibodies (Cheng *et al.*^2017^; Zhen *et al.*^2017^) was not observed in non-human primates treated with an IFNα antagonist (Nganou-Makamdop *et al.*^2018^). Recent studies demonstrated that virus control is mediated by IFNβ and T cell exhaustion by IFNα in murine LCMV infection (Ng *et al.*^2015^); and that in humanized HIV-infected mice, IFNβ or IFNα14 but not the commonly used IFNαa2 significantly suppressed HIV replication (Abraham *et al.*^2016^; Lavender *et al.*^2016^). Therefore, it is tempting to speculate that different members of the IFN-I family play different roles and manipulating specific type I IFNs might be needed to selectively target detrimental activities while maintaining beneficial ones.

### Overall Outcome and Implication for HIV Infection

All HIV/SIV studies on type I IFNs undoubtedly concur that the effects of enhancing or suppressing IFN-I signaling are strongly influenced by the timing of treatment (acute or chronic infection). During acute infection, the antiviral effects of IFN-I outweigh the deleterious effects. IFN-I treatment has so far not been shown to match the efficacy reached with ART and would not be useful as monotherapy. However, ART alone does not resolve chronic immune activation (Fernandez *et al.*^2011^; Hunt 2012; Dunham *et al.*^2014^; Sereti *et al.*^2017^) and is not sufficient to completely purge the HIV reservoir (Henrich *et al.*^2017^), potentially because antiretroviral drugs do not reach sufficient levels in lymphoid tissues (Fletcher *et al.*^2014^) where HIV predominantly resides (Rothenberger *et al.*^2015^; Lorenzo-Redondo *et al.*^2016^). Because IFN-I readily penetrates tissues (Johns *et al.*^1990^), a legitimate question to address would be whether ART and IFN-I combination in acute HIV infection may impact the establishment or size of the virus reservoir. During chronic HIV, limiting IFN-I’s contribution to ongoing immune activation is thought to be a major target for clinical improvement. To date, this concept remains to be proven in experimental settings. Importantly, no study in non-human primate or humanized mice has so far shown a detrimental effect of blocking IFN-I signaling during chronic HIV/SIV infection; suggesting that type I IFNs may not be as critical for the control of the infection as they are in the acute phase.

A better understanding of the complex roles of IFN-I in HIV infection is likely to be achieved by addressing the following understudied questions:

- Are all ISGs equally important to disease progression? In other words, do some of the many ISGs upregulated during acute infection or maintained during chronic infection associate with clinical outcome? Could these help predict disease course?
- What are the major differences in IFN-I signaling or pathways induced by the various IFN-I types and could these help understand emerging data on their functional diversity in the control of HIV infection?
- Can an optimal balance of maximal upregulation of restriction factors and minimal upregulation of immune activating ISGs be achieved? Can this help reduce the virus reservoir?
- Can IFN-I (sub)type-specific targeting in chronic infection reduce systemic immune activation thereby ameliorating clinical outcome?

Noteworthy parallels exist between studies on HIV/SIV infection and studies on other viral infections. For instance, the paradoxical role of IFN-I in inducing antiviral responses and enhancing pathology has been described for influenza (Garcia-Sastre and Biron 2006; Davidson *et al.*^2014^) and SARS (Huang *et al.*^2005^). Taken together, data from several studies suggest that IFN-I signaling is essential to cytokine responses and recruitment of immune cells, which in turn can promote pathology. In influenza, administration of an agonist to the lipid metabolite sphingosine 1 phosphate receptor (S1P1R) expressed on pDCs was shown to suppress IFN-I signaling in mice and human cells and blunt proinflammatory responses during influenza infection in mice that showed improved survival (Teijaro *et al.*^2011^; Walsh *et al.*^2011^; Teijaro *et al.*^2016^). Thus, provided that optimal conditions are met, it may indeed be possible to manipulate IFN-I signaling to improve clinical outcome in HIV infection.
